# GTL001, a bivalent therapeutic vaccine against human papillomavirus 16 and 18, induces antigen-specific CD8^+^ T cell responses leading to tumor regression

**DOI:** 10.1371/journal.pone.0174038

**Published:** 2017-03-16

**Authors:** Michaël Esquerré, Myriam Bouillette-Marussig, Anne Goubier, Marie Momot, Christophe Gonindard, Hélène Keller, Astrid Navarro, Marie-Christine Bissery

**Affiliations:** 1 Genticel, Labège, France; 2 Genticel, Paris, France; Penn State University School of Medicine, UNITED STATES

## Abstract

**Background:**

Prophylactic vaccines are available for women and girls not yet infected with HPV, but women already infected with HPV need a treatment to prevent progression to high-grade cervical lesions and cancer. GTL001 is a bivalent therapeutic vaccine for eradicating HPV-infected cells that contains HPV16 E7 and HPV18 E7 both fused to detoxified adenylate cyclase from *Bordetella pertussis*, which binds specifically to CD11b^+^ antigen-presenting cells. This study examined the ability of therapeutic vaccination with GTL001 adjuvanted with topical imiquimod cream to induce functional HPV16 E7- and HPV18 E7-specific CD8^+^ T cell responses.

**Methods:**

Binding of GTL001 to human CD11b was assessed by a cell-based competition binding assay. Cellular immunogenicity of intradermal vaccination with GTL001 was assessed in C57BL/6 mice by enzyme-linked immunospot assay and *in vivo* killing assays. *In vivo* efficacy of GTL001 vaccination was investigated in the TC-1 murine HPV16 E7-expressing tumor model.

**Results:**

GTL001 bound specifically to the human CD11b/CD18 receptor. GTL001 adjuvanted with topical 5% imiquimod cream induced HPV16 E7 and HPV18 E7-specific CD8^+^ T cell responses. This CD8^+^ T-cell response mediated *in vivo* killing of HPV E7-expressing cells. In the HPV16 E7-expressing tumor model, GTL001 adjuvanted with imiquimod but not imiquimod alone or a combination of unconjugated HPV16 E7 and HPV18 E7 caused complete tumor regression.

**Conclusions:**

GTL001 adjuvanted with topical 5% imiquimod is immunogenic and induces HPV16 E7 and HPV18 E7-specific CD8^+^ T cell responses that can kill HPV E7-expressing cells and eliminate HPV E7-expressing tumors.

## Introduction

Nearly all cervical cancer is caused by human papillomavirus (HPV) [1]. B cell-mediated immunity to the viral capsid proteins L1 and L2 can provide efficient protection against HPV infection [2], but it is not effective for patients who already are infected with HPV [3, 4]. HPV-infected women with normal cervical cytology or mild abnormalities currently have no treatment options other than watchful waiting for HPV clearance or, if high-grade lesions or cancer develop, surgery with possible adjunct chemotherapy [5]. Although surgical excision is very successful for high-grade cervical lesions and cervical cancer, it can lead to infertility, pregnancy problems, incontinence, and sexual dysfunction [5–8]. Women infected with HPV who still have normal cervical cytology or only mild or borderline abnormalities need a treatment that can prevent their infections from progressing to cervical lesions and cancer.

A therapeutic HPV vaccine could offer an opportunity to clear HPV infections before local immunosuppression and viral escape mechanisms develop [5, 9]. To control existing infections and malignancies, a therapeutic vaccine should induce antigen-specific T-cell-mediated immunity against HPV-infected cells. Several therapeutic vaccines are in development and have been tested in human clinical trials [10]. Most target the HPV E7 protein and, in some cases, also the E6 protein, which are continuously expressed during the viral cycle, are required for cellular transformation, and are expressed by all cervical cancers and precursor lesions [11–13]. Some of these candidate vaccines have shown promise in treating vulvar and high-grade cervical intraepithelial neoplasia, and some induce cellular immunity against HPV, but they have only been tested in women who already have high-grade lesions [14–16].

GTL001 is a bivalent therapeutic vaccine for preventing cervical cancer in women who have been infected with HPV16 or HPV18 and who still have normal cervical cytology or mild or borderline abnormalities. GTL001 is designed to promote clearance of HPV16 and HPV18 infections before advanced lesions develop. GTL001 contains HPV16 and HPV18 E7, each fused to detoxified adenylate cyclase from *Bordetella pertussis* (CyaA) as a vaccine vector. The N-terminus of CyaA is translocated into host cells by binding to CD11b [17]. This unique property can be harnessed to specifically deliver viral or tumor antigens to CD11b^+^ antigen-presenting cells (APC) and thereby induce antigen-specific CD4^+^ and CD8^+^ T cell responses against these antigens. In mice, intradermal vaccination with a recombinant CyaA bearing the HPV16 E7 antigen has been shown to induce a HPV16 E7-specific T cell response and, when adjuvanted with CpG oligodeoxynucleotide (ODN) 1826, a toll-like receptor (TLR) 9 agonist, to eliminate established HPV16 E7-expressing tumors [18, 19]. Two formulations of GTL001, a solution formulation and a more concentrated powder formulation, adjuvanted with topical imiquimod cream, have been tested in a phase I clinical trial, and both were safe and induced E7-specific CD8^+^ T cell responses [20]. Due to these promising results, the powder formulation is currently being tested in a phase II clinical trial (NCT01957878).

Here, we describe the ability of intradermal vaccination with GTL001 to induce both HPV16 E7- and HPV18 E7-specific CD8^+^ T cell responses in C57BL/6 mice. We also explore whether the HPV E7-specific T cells induced by GTL001 are functional cytotoxic CD8^+^ T lymphocytes that can promote the regression of HPV16 E7-expressing tumors.

## Materials and methods

### Cells

CHO-CR3 cells, a Chinese hamster ovary cell line transduced to express the human CD11b/CD18 receptor, was obtained from Douglas Golenbock (University of Massachusetts Medical School, Worcester, MA, USA) and was enriched by positive selection using CD11b magnetic beads (Miltenyi Biotec, Köln, Germany). These CD11b^+^ cells were cultured in Ham’s F12 medium supplemented with 10% heat-inactivated fetal bovine serum (Sigma-Aldrich, St. Louis, MO, USA), 500 μg/mL G418, 100 units/mL penicillin, and 100 μg/mL streptomycin. HPV16 E7-expressing TC-1 cells from American Type Culture Collection (Manassas, VA, USA) were grown in RPMI 1640 medium supplemented with 10% heat-inactivated fetal bovine serum, 400 μg/mL G418, 100 units/ml penicillin, and 100 μg/ml streptomycin. Murine splenocytes were cultured in complete medium (RPMI 1640 medium supplemented with 10% heat-inactivated fetal bovine serum, 100 units/ml penicillin, 100 μg/ml streptomycin, 1 μM beta-mercaptoethanol, 1x non-essential amino acids [LONZA, Basel, Switzerland], and 1 mM sodium pyruvate [LONZA]).

### Animals

C57BL/6 female mice (6 to 10 weeks old) were obtained from Janvier (Le Genest-St-Isle, France) and Charles River (L’Arbresles, France). The mice were housed under specific pathogen-free conditions and handled according to protocols approved by the Ethics Committee on Animal Experimentation (registration no. 122) and in compliance with European Union directive 2010/63/UE. Mice were fed type RM1 food (Special Diet Services, Essex, UK) and sterile-filtered tap water ad libitum. The studies were carried out either at Genticel (Labège, France) or at Oncodesign (Dijon, France) in compliance with the European Union directive 2010/63/EU for animal experiments. Animal care was in accordance with institutional guidelines. The study protocols were approved by the respective institutional animal care and use committees: for Genticel, by the *Comité d’Ethique en matière d’Expérimentation Animale* (*Unité Inserm 006/Centre Régional d’Exploration Fonctionnelle et Ressources Expérimentales*), under CNREEA number C2EA-122; and for Oncodesign, by their internal ethics committee (OncomEt), under CNREEA number 91. Mouse viability and behavior were recorded daily, and body weight was measured once or twice weekly. Mice were euthanized by cervical dislocation under anesthesia if they lost 15% of their body weight for 3 consecutive days or 20% for 1 day or if they displayed signs of suffering (cachexia, weakening) or compound toxicity (hunching, convulsions). In addition, mice included in the TC-1 tumor regression model were euthanized if their tumors reached 10% of the body weight or interfered with movement or feeding.

### GTL001

GTL001 consists of equal amounts of two recombinant HPV E7-CyaA fusion proteins expressed in and purified from *Escherichia coli*. The HPV16 E7-CyaA fusion protein included amino acids 1–29 of HPV16 E7 inserted between amino acids 319 and 320 of CyaA and amino acids 43–98 of HPV16 E7 inserted between amino acids 224 and 235 of CyaA. The HPV18 E7-CyaA fusion protein included amino acid 1–31 of HPV18 E7 inserted between amino acids 319 and 320 of CyaA and amino acids 43–105 of HPV18 E7 inserted between amino acids 224 and 235 of CyaA [19]. The solution formulation of GTL001 was 0.5 μg/μL in PBS with 1.83 M urea. The powder formulation of GTL001 was reconstituted prior to use with sterile water to 2.5 μg/μL. Both formulations were confirmed to be endotoxin-free.

### Adjuvant, reagents, and peptides

Topical 5% imiquimod cream (Aldara^™^) was from 3M Health Care Limited (Loughborough, UK). TO-PRO-3 and RPMI-Glutamax, 5-(and-6)-carboxyfluorescein succinimidyl ester (CFSE) were obtained from Thermo Fisher Scientific (Waltham, MA, USA). Empty CyaA was produced in *Escherichia coli* as described previously [19]. CyaA was biotinylated with EZ-Link^®^ HPDP-Biotin (Thermo Fisher Scientific) and purified with a 7000-Da molecular weight cutoff Zeba^™^ Spin desalting column (Thermo Fisher Scientific). Streptavidin-phycoerythrin (PE) was from BD Biosciences (Franklin Lakes, NJ, USA). Synthetic peptides HPV16 E7_49-57_, HPV18 E7_AS43-49_, and ovalbumin_257-264_ were obtained from Millegene (Toulouse, France), 15-mer peptide banks (HPV16 E7 and HPV18 E7) overlapping by 11 amino acids were from Eurogentec (Seraing, Belgium). HPV16 E7_43-98_ was synthesized by Genosphere (Paris, France).

### In vitro binding of CyaA to human monocytes and lymphocytes

Human peripheral blood mononuclear cells (PBMC) were isolated from the blood of three healthy donors (Etablissement Français du Sang, Rungis, France) by Ficoll density centrifugation and frozen in liquid nitrogen. Thawed PBMC in RPMI1640 cell culture medium supplemented with 10% fetal calf serum (0.5 to 1 × 10^6^ cells) were incubated with BluVid (Molecular Probes-Thermo Fisher, Eugene, OR) for 30 min on ice, washed with PBS, and suspended in binding buffer (0.5% glucose, 140 mM NaCl, 2 mM CaCl_2_, 2 mM MgCl_2_, 10 mM HEPES, and 1% fetal bovine serum). The cells were then mixed with binding buffer with or without 50 μg/ml biotinylated CyaA-HPV16 E7 for 30 min on ice. After washing, the cells were mixed with one of the following combinations of mouse monoclonal antibodies (mAbs) in binding buffer: (a) peridinin-chlorophyll-protein (PerCP)-labeled anti-CD11c mAb (marker of dendritic cells; Abcam, Cambridge, UK), FITC-labeled anti-CD14 mAb (marker of monocytes; BD Biosciences), and allophycocyanin (APC)-labeled anti-human CD20 mAb (marker of B lymphocytes; BD Biosciences); (b) Alexa Fluor^®^ 700-labeled anti-human CD11b mAb (marker of interest; BD Biosciences), PerCP-labeled anti-CD11c, FITC-labeled anti-CD14 mAb (marker of monocytes), and APC-labeled anti-CD20 mAb (marker of B lymphocytes); (c) fluorescently labeled isotype controls (PerCP-labeled IgG1, FITC-labeled IgG2a, APC-labeled IgG2b, and Alexa Fluor^®^ 700-labeled-IgG1; all from BD Biosciences); or (d) PE-labeled streptavidin (BD Biosciences). After washing, cells were fixed with Cell Fix (BD Biosciences). Fluorescence was detected by flow cytometry using a FACSCanto A (BD Biosciences). Fluorescence in viable cells (BluVid-negative) was analyzed using FlowJo version 9.2 (FlowJo, Ashland, OR) after gating on monocytic and lymphocytic populations as determined by their FSC/SSC parameters.

### In vitro CD11b competition binding assay

The experiments used CHO-CR3 cells transduced to express human CD11b (CHO-CR3 CD11b^+^ cells). CHO-CR3 CD11b^+^ cells were incubated for 30 min on ice in binding buffer with 40 nM biotinylated CyaA-HPV16 E7 and 0 to 512 nM CyaA, 0 to 512 nM test substance, or 512 nM heat-inactivated GTL001. After washing, cells were incubated for 30 min on ice with 0.5 mg/mL PE-labeled streptavidin. Cells were re-suspended in 10 nM TO-PRO-3. Bound biotin-CyaA-HPV16 E7 was detected by flow cytometry using a FACSCanto A equipped with a high-throughput sampler (BD Biosciences). Analyses were performed after gating on living cells as based on exclusion of TO-PRO-3 positive cells. The dose-response curves were fitted using Prism (GraphPad Software, La Jolla, CA, USA) to estimate the concentration resulting in 50% inhibition (IC_50_).

### T cell responses in GTL001-immunized mice

C57BL/6 female mice (6 to 10 weeks old) were acclimated for 1 week before vaccination. Mice (5/group) were anesthetized and vaccinated intradermally in the inner ear with GTL001 (10 μg) or placebo (PBS with 1.83 M urea). Immediately and 24 h after vaccination, 25 mg of 5% imiquimod cream was applied at injection sites (ear skin on both ears) by rubbing in for at least 15 s using a sterile cone until complete penetration. After 7 days, mice were anesthetized and sacrificed by CO_2_ inhalation or cervical dislocation. Spleens were collected and homogenized in complete medium. Splenocytes were isolated by centrifugation for 20 min at 1500 × g though Lymphocyte-M separation medium (Cedarlane, Burlington, Ontario, Canada). T-cell responses were measured by interferon-γ (IFN-γ) enzyme-linked immunospot (ELISpot) and cytometric bead array, as described below.

### IFN-γ ELISpot

In triplicate wells of 96-well plates, pooled splenocytes from GTL001-immunized or placebo-injected mice (1 × 10^6^ cells in 100 μL complete medium) were mixed with 100 μL of complete medium containing 1 μg/mL of major histocompatibility complex (MHC) class I (H-2^b^)-restricted peptides (HPV16 E7_49-57_, HPV18 E7_AS43-49_, or ovalbumin_257-264_), no addition (negative control), or 1 μg/ml hamster anti-mouse CD3_Ɛ_ antibody (BD Biosciences; positive control for polyclonal T-cell responsiveness). After 20 h at 37°C, frequencies of IFN-γ-secreting CD8^+^ T cells were assessed using a mouse IFN-γ ELISpot PLUS kit according to the manufacturer’s instructions (Mabtech, Nacka Strand, Sweden). Spot-forming cells were counted using a Bioreader 5000 Pro S plate reader (Biosys, Karben, Germany).

### Analysis of cytokine secretion by cytometric bead array

Splenocytes from GTL001-immunized or placebo-injected mice (4 × 10^6^ in 2 ml) were cultured in 24-well plates and stimulated in the presence or absence of 30 μg/mL synthetic HPV16 E7_43-98_. Culture supernatants were harvested after 3 days and stored at −20°C until use. These supernatants were assayed for the presence of IFN-γ, interleukin (IL)-6, tumor necrosis factor-α (TNF-α), and IL-4 by BD™ Cytometric Bead Array (BD Biosciences) according to the manufacturer’s instructions, measured by flow cytometry using a FACSCanto (BD Biosciences). Results were analyzed using FCAP Array™ software (Soft Flow, Pecs, Hungary).

### *In vivo* killing assay

Splenocytes (5 × 10^6^ cells/ml) from naïve C57BL/6 mice were pulsed for 3 h at 37°C in complete medium containing no addition or a combination of 3 μg/ml of HPV16 E7_49-57_ and 3 μg/ml of HPV18 E7_AS43-49_ peptides. Cells were then collected and washed with PBS. Unpulsed target cells were treated for 10 min at 37°C with 0.1 μM CFSE (CFSE^low^) and with 1 μM (CFSE^high^) for HPV E7-pulsed target cells. The CFSE^low^ and CFSE^high^ cells were mixed at a 1:1 ratio, washed twice in PBS + 2% fetal bovine serum, and suspended in PBS at 100 × 10^6^ cells/ml for adoptive transfer to recipient mice vaccinated with either GTL001 + 5% imiquimod cream or placebo + 5% imiquimod cream. At day 6, recipient mice were anaesthetized and intravenously injected with 100 μL of the target cell mixture via the retro-orbital venous sinus. The next day, mice were anesthetized and killed under anesthesia by CO_2_ inhalation or cervical dislocation. Spleens were collected and homogenized in complete medium, after which cells were washed with PBS + 1% fetal bovine serum. Target cells were tracked *ex vivo* in individual mice by flow cytometry using a FACSCanto A (BD Biosciences) and analyzed using FlowJo software (Ashland, OR, USA) with exclusion of dead cells based on forward scatter-side scatter criteria. The percentage of *in vivo* killing was calculated as described previously [21]: % killing = 100 − ([(% peptide pulsed in vaccinated/% unpulsed in vaccinated)/(% peptide pulsed before injection/% unpulsed before injection)] × 100).

### TC-1 tumor regression model

C57BL/6 mice were injected subcutaneously in the flank with 1 × 10^6^ TC-1 cells in 200 μL of PBS. The mice (10 or 11 per group) were therapeutically vaccinated on day 11 and again on day 39 by the intradermal route in the inner ear with 10 μg of GTL001. Immediately and 24 h after each vaccination, 25 mg of 5% imiquimod cream was rubbed in at the injection site for at least 15 s using a sterile cone until complete penetration. Tumors were measured with calipers twice weekly between day 5 and 60. Mice were euthanized under anesthesia when their tumors reached a volume of 2000 mm^3^ or 60 days after tumor induction.

### Statistical analysis

Statistical analysis was made using Systat (Systat Software, San Jose, CA, USA) and R (Revolution Analytics, Redmond, WA, USA). Cell survival in the in vivo killing assay for treatment vs. placebo was compared by Newman-Keuls test. In cases where the treatment effect was significant, p-values were calculated using a Fisher Exact test. A p-value below 0.05 was considered statistically significant.

## Results

### GTL001 specifically targets CD11b

**Fig 1:**
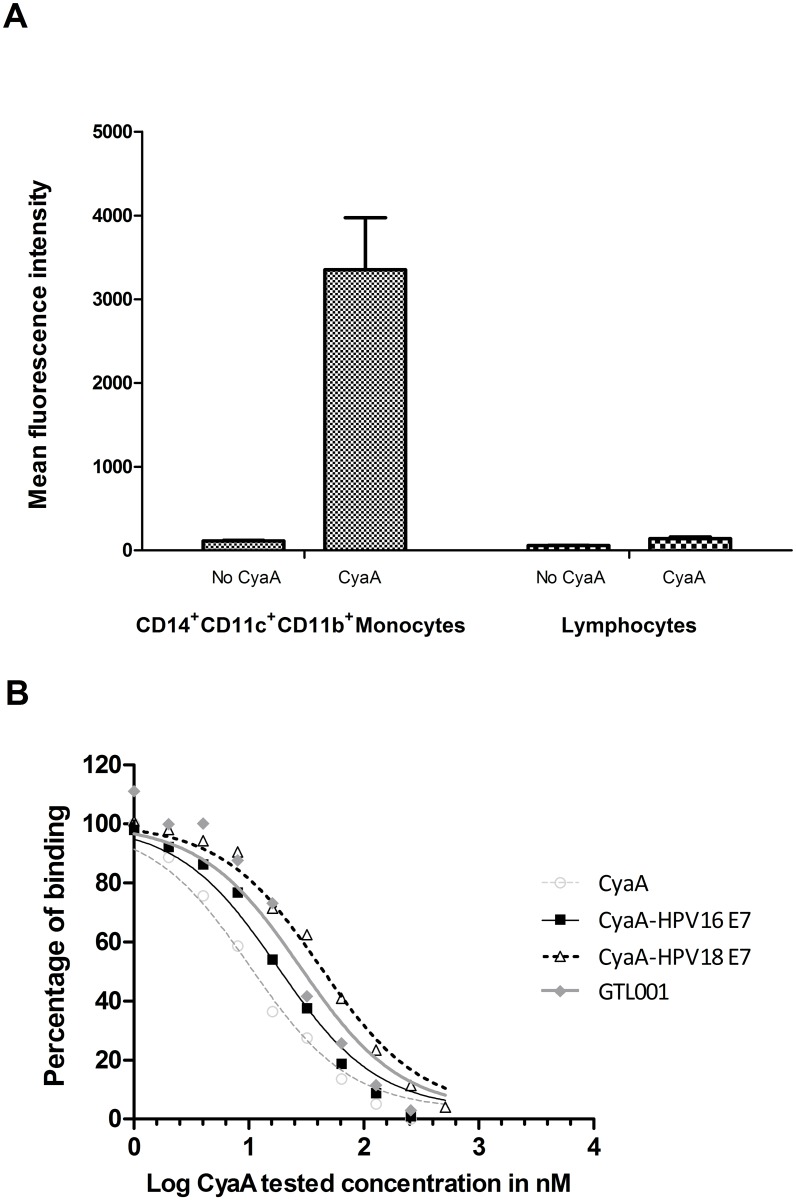
GTL001 binding to the human CD11b/CD18 receptor. (A) Human PBMC were incubated with or without biotinylated CyaA-HPV16 E7, followed by fluorescently labeled antibodies. Bound biotinylated CyaA-HPV16 E7 was detected with PE-streptavidin and analyzed by flow cytometry. Results are the means of triplicate determinations ± standard deviation. (B) CHO-CR3 CD11b^+^ cells were incubated with biotinylated CyaA-HPV16 E7 and 0 to 512 nM CyaA, the solution formulation of GTL001, CyaA-HPV16 E7, or CyaA-HPV18 E7. After washing, bound biotinylated CyaA was detected with PE-labeled streptavidin and flow cytometry. Results are from a single experiment in which all substances were compared but are representative of two to six experiments per tested substance.

CyaA was used as a vaccine vector because it binds specifically to human and mouse CD11b expressed on APC [17, 22]. In an initial experiment, we found that biotinylated CyaA-HPV16 E7 binds to human CD14^+^ CD11b^+^ CD11c^+^ monocytes (i.e. dendritic cells) but very poorly to human lymphocytes (Fig 1A). Biotinylated CyaA-HPV16 E7 also bound to DC2.4 cells, a murine dendritic cell line (data not shown).

We further examined the specific binding of GTL001 to CD11b by a competition binding assay using CHO cells transduced to express human CD11b. In this experiment, binding of biotinylated CyaA-HPV16 E7 to human CD11b on CD11b^+^ cells was competed by CyaA-HPV16 E7 (IC_50_ = 18 nM), CyaA-HPV18 E7 (IC_50_ = 42 nM), the solution formulation of GTL001 (IC_50_ = 28 nM), and empty CyaA (IC_50_ = 10 nM) (Fig 1B). Binding of biotinylated CyaA-HPV16 E7 to CD11b^+^ cells was not competed by 512 nM heat-inactivated GTL001, and furthermore, biotinylated CyaA-HPV16 E7 did not bind to CD11b^-^ cells (data not shown), confirming specific binding to CD11b. Therefore, inserting HPV16 E7 or HPV18 E7 sequences in CyaA did not eliminate recognition by CD11b.

### GTL001 adjuvanted with 5% imiquimod cream induces HPV E7-specific CD8^+^ T cell responses in a bivalent fashion

We next examined the ability of GTL001 to induce HPV16 E7- and HPV18 E7-specific CD8^+^ T cell responses. C57BL/6 mice were vaccinated intradermally with placebo or 10 μg of GTL001. Immediately and 24 h after vaccination, topical 5% imiquimod cream was applied as an adjuvant at injection sites to promote an antigen-specific CD8^+^ T cell response [23]. Splenocytes isolated from immunized mice were stimulated with HPV16 E7_49-57_ or HPV18 E7_AS43-49_. HPV16 E7_49-57_ represents the most potent H-2^b^-restricted HPV16 E7 epitope [24]. HPV18 E7_AS43-49_ is a neo-epitope at the junction of Ala-Ser from the CyaA sequence and the E7_43-49_ sequence, and it is the only MHC class I restricted HPV18 E7 epitope in the C57BL/6 genetic background [25].

**Fig 2:**
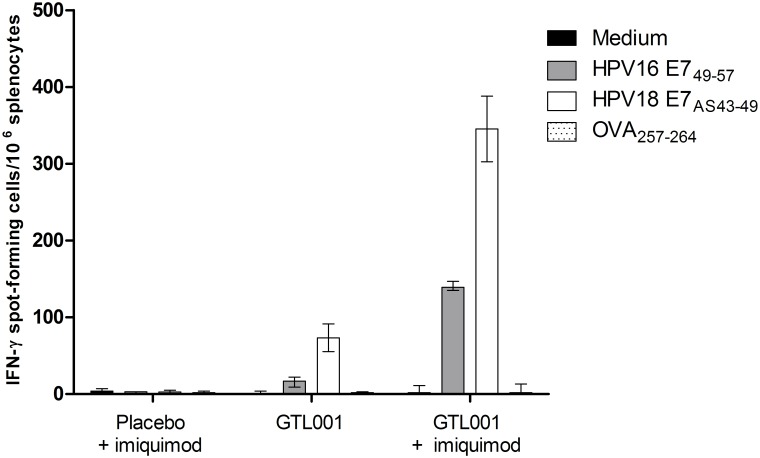
Induction of HPV E7-specific CD8^+^ T-cell responses as measured by ELISpot. C57BL/6 mice (5/group) were vaccinated intradermally with 10 μg of the solution formulation of GTL001 or placebo (PBS with 1.83 M urea) on day 0. Immediately and 24 h after vaccination, 25 mg of 5% imiquimod cream/ear was applied at each vaccination site. On day 7, splenocytes from each group were collected, pooled, and stimulated *ex vivo* with medium alone or with the H-2^b^ restricted epitopes of HPV16 E7 (HPV16 E7_49-57_), HPV18 E7 (HPV18 E7_AS43-49_), or ovalbumin (OVA_257-264_). After 20 h, T-cell responses were assessed by IFN-γ ELISpot. Values represent means of three determinations per splenocyte pool ± standard deviations.

As measured by IFN-γ ELISpot, mice vaccinated with the solution formulation of GTL001 developed both HPV16 E7- and HPV18 E7-specific CD8^+^ T cell responses (Fig 2). Adjuvantation with topical 5% imiquimod cream increased these GTL001-induced HPV E7-specific CD8^+^ T cell responses. Similar results were found using the powder formulation of GTL001 (data not shown).

**Fig 3:**
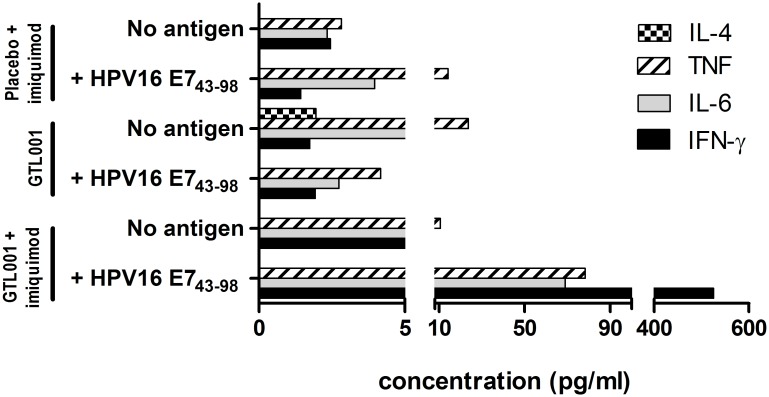
Induction of HPV E7-specific CD8^+^ T-cell responses as measured by cytometric bead array. C57BL/6 mice (5/group) were vaccinated intradermally with 10 μg of the solution formulation of GTL001 or placebo (PBS with 1.83 M urea) on day 0. Immediately and 24 h after vaccination, 25 mg of 5% imiquimod cream/ear was applied at each vaccination site as indicated. Splenocytes were collected on day 7, pooled, and stimulated *ex vivo* without or with 30 μg/mL synthetic HPV16 E7 antigen (HPV16 E7_43-98_). After 3 days, production of IFN-γ, IL-6, TNF-α, and IL-4 was assessed by cytometric bead array. Values are from single determinations per splenocyte pool. IL-4 does not appear in several cases because it was undetectable.

Cytometric bead array further showed that GTL001 adjuvanted with 5% imiquimod cream induced the *ex vivo* production of T_H_1 cytokines (IFN-γ, IL-6, and TNF-α) (Fig 3). These cytokines were not detected after antigen-specific re-stimulation of total splenocytes from mice vaccinated with placebo + 5% imiquimod cream or with GTL001 alone.

**Fig 4:**
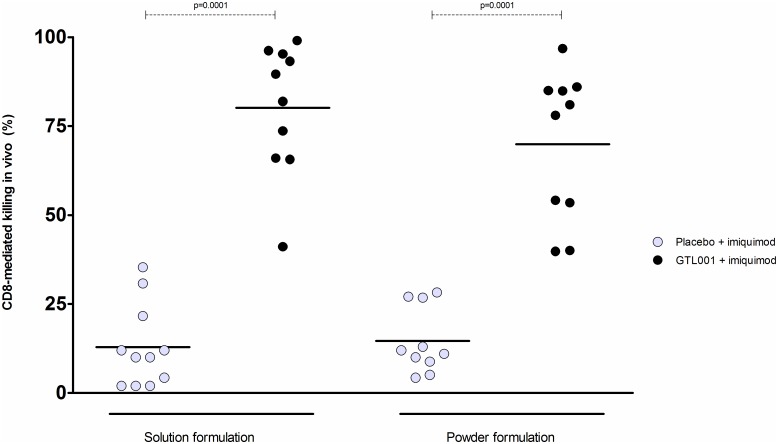
*In vivo* killing assay. C57BL/6 mice were vaccinated intradermally with 10 μg of the solution or powder formulation of GTL001 on day 0. Immediately and 24 h after vaccination, 25 mg of 5% imiquimod cream/ear was applied at each vaccination site. On day 6, the mice were injected via the retro-orbital venous sinus with CFSE-labeled splenocytes from naïve C57BL/6 mice that had been pulsed with MHC class I/H-2^b^ restricted peptides of HPV16 and HPV18 E7 (HPV16 E7_49-57_ and HPV18 E7_AS43-49_) or no addition. Splenocytes were collected the next day (day 7) and analyzed by flow cytometry to measure antigen-specific CD8^+^ T cell-mediated killing. Each dot represents the percent CD8-mediated *in vivo* killing for an individual mouse. Horizontal bars indicate means for each group of mice. Data were pooled from 3 independent experiments. P-values were determined by Newman-Keuls test.

The functional activity of GTL001-induced HPV E7-specific CD8^+^ T cells was assessed by an *in vivo* killing assay. Splenocytes from naïve donor C57BL/6 mice were pulsed with the H-2^b^-restricted peptides of HPV16 E7 and HPV18 E7 or left unpulsed to generate syngeneic target cells. The solution and powder formulations of GTL001, adjuvanted with topical 5% imiquimod cream, equally induced HPV E7-specific cytotoxic T lymphocytes (CTL) able to eliminate syngeneic target cells (Fig 4).

### Therapeutic vaccination with GTL001 adjuvanted with 5% imiquimod cream eradicates HPV16 E7-expressing tumors in C57BL/6 mice

**Fig 5:**
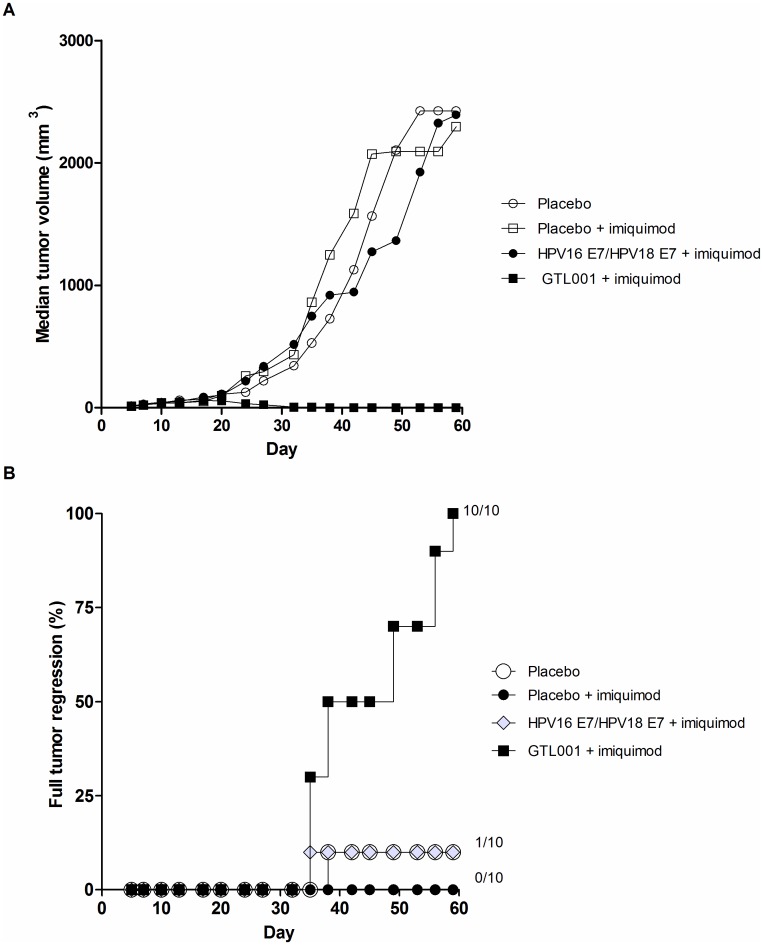
Induction of tumor regression by GTL001. Solid tumors were induced by subcutaneous injection of C57BL/6 mice with HPV16 E7-expressing TC-1 cells. On day 11, when tumors were at least palpable, and again on day 39, mice (10/group) were vaccinated intradermally with 10 μg of the solution formulation of GTL001, an equimolar amount of unconjugated HPV16 E7 and HPV18 E7 proteins, or placebo (PBS with 1.83 M urea). Where indicated, mice also received 25 mg imiquimod cream/ear at the vaccination site immediately and 24 h after each vaccination (days 11, 12, 39, and 40). Tumor size was measured every 2 to 3 days. (A) Median tumor volume. (B) Percent full tumor regression.

We investigated the potential therapeutic effect of GTL001 in a preclinical murine model of HPV E7-expressing tumors. Mice were inoculated with TC-1 tumor cells expressing the HPV16 E7 oncoprotein, in which E7 is necessary for tumor growth [26]. After 11 days, when tumors were at least palpable, mice were vaccinated with GTL001 solution, an equimolar amount of unconjugated HPV16 E7 and HPV18 E7 protein (i.e. lacking the CyaA vector), or placebo and adjuvanted with topical 5% imiquimod cream. Mice received a second vaccination 28 days later (day 39), and tumor regression was assessed until day 60. At the end of the clinical monitoring, no tumors were detected in mice treated with GTL001 solution + imiquimod, whereas mice treated with placebo alone, placebo + imiquimod cream, or a combination of unconjugated HPV16 E7 and HPV18 E7 protein rapidly grew tumors, all at approximately the same rate and to the same extent (Fig 5A). At the end of the study, all 10 mice treated with GTL001 + imiquimod were tumor-free, whereas only 1 of 10 of mice treated with placebo, 1 of 10 treated with unconjugated HPV16 E7 and HPV18 E7, and 0 of 10 mice treated with placebo + imiquimod cream were tumor-free (Fig 5B).

**Fig 6:**
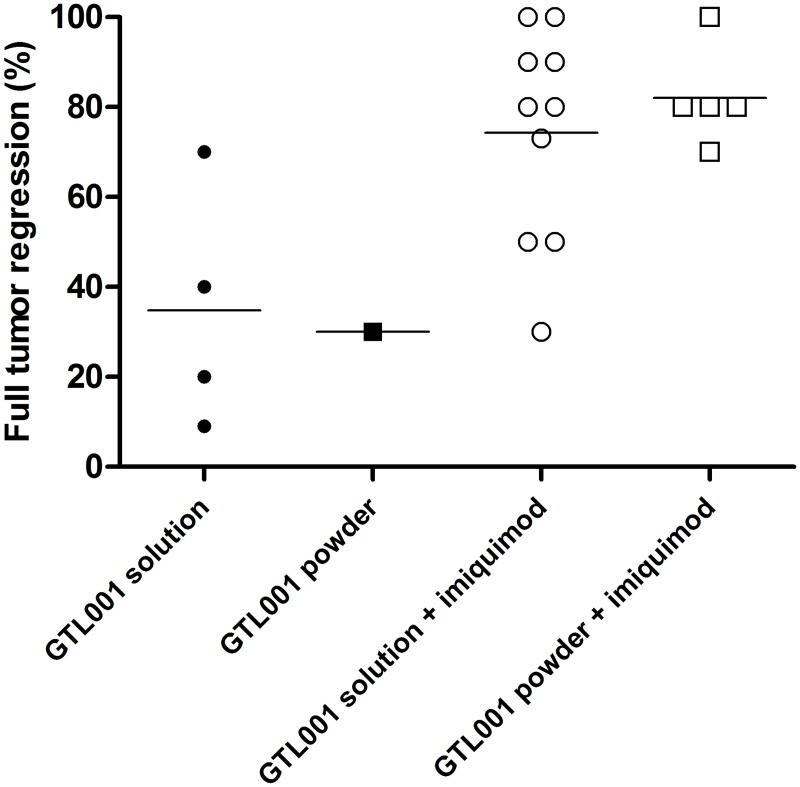
Adjuvant effect of imiquimod on induction of tumor regression and promotion of survival by GTL001. Solid tumors were induced by subcutaneous injection of C57BL/6 mice with HPV16 E7-expressing TC-1 cells. On day 11, when tumors were at least palpable, and again on day 39, mice (10/group) were vaccinated intradermally with 10 μg of the solution or powder formulation of GTL001. Where indicated, mice also received 25 mg imiquimod cream/ear at the vaccination site immediately and 24 h after each vaccination (days 11, 12, 39, and 40). Symbols indicate % full tumor regression for each group of 10 mice, and horizontal bars indicate means.

In a separate experiment, we evaluated the adjuvant effect of imiquimod in the same murine model of HPV16 E7-expressing tumors (Fig 6). At day 60, when combined with imiquimod, both the solution and powder formulations of GTL001 substantially and significantly promoted full tumor regression and survival compared to the same formulation of GTL001 alone. These results confirm that imiquimod is necessary for GTL001 to have an optimal effect on HPV16 E7-expressing tumors.

## Discussion

The catalytic domain of *B*. *pertussis* CyaA translocates into the cytosol of host cells by binding to CD11b [17]. This unique property is independent of enzymatic activity and can be harnessed to specifically deliver viral or tumor antigens to CD11b^+^ APC. Upon binding to CD11b on APCs, CyaA fusion proteins deliver their fusion partners to the cytosol, where they are efficiently processed and presented on MHC molecules, resulting in strong, long-lasting CTL responses [22, 27, 28].

Preville *et al*. showed that CyaA fusion proteins containing the full sequence or various subfragments of the HPV16 E7 protein can induce specific T_H_1 and CTL responses in C57BL/6 mice [19]. They also showed that in all mice grafted with HPV16 E7-expressing TC-1 tumor cells, CyaA-HPV16 E7_Δ30–42_, when adjuvanted with CpG ODN 1826, triggers complete tumor regression. CyaA fusion proteins harboring human melanoma epitopes have also been shown to be efficiently processed by human dendritic cells and to activate epitope-specific CTL clones [27].

In the current study, we examined GTL001, a candidate HPV therapeutic bivalent vaccine consisting of equal amounts of CyaA-HPV16 E7 and CyaA-HPV18 E7 fusion proteins. GTL001 bound specifically to CD11b via the CyaA portion of the fusion protein and, adjuvanted with topical 5% imiquimod cream, induced CD8^+^ T-cell responses specific to both HPV16 and HPV18 E7. These findings extend the results of Preville *et al*. [19] by showing that GTL001 induces parallel CD8^+^ T cell responses to both HPV16 and HPV18 E7, both of which can mediate killing of HPV E7-expressing cells. Thus, this study demonstrates the multivalent potential of CyaA technology as a vaccine vector. Our findings also show that topical imiquimod cream, like CpG ODN 1826, is an effective adjuvant for CyaA-based therapeutic vaccines. Importantly, when adjuvanted with imiquimod cream, GTL001 but not unconjugated E7 proteins induced complete regression of HPV16 E7-expressing TC-1 tumors. Further, this is the first study to show the induction of a HPV18 E7-specific T cell response in a mouse model, although we were not able to confirm the ability of GTL001 to induce regression of HPV18 E7-expressing tumors due to the lack of a commercially available HPV18 E7-expressing mouse tumor model.

This study focused on the mechanism of action of GTL001 adjuvanted with topical 5% imiquimod cream, as used in the completed phase I [20] and current phase II (NCT01957878) clinical trials. Studies in mice and in humans have demonstrated that 5% imiquimod cream is a potent adjuvant of T-cell-based therapeutic vaccines [29, 30]. Imiquimod stimulates APC by binding to TLR7 and TLR8, which enhances the production of pro-inflammatory cytokines and chemokines and promotes dendritic cell maturation [23]. CpG ODN 1826, used in the study by Preville *et al*. [19], works in a similar way, although via TLR9 [31]. Because TLR agonists like imiquimod are T_H_1-cell-polarizing, they are ideal adjuvants for CyaA-based therapeutic vaccines intended for treating cancer and viral infections [32, 33]. We have selected topical administration of imiquimod for clinical use because it promotes human dermal dendritic cell maturation and CD8^+^ T-cell cross-priming, which is not the case when imiquimod is administered intradermally [34]. Also, 5% imiquimod cream is already licensed as a topical agent to treat genital warts, although it is used more frequently (three times/week) and for longer (16 weeks) as a stand-alone therapy than as an adjuvant as for T cell-based therapeutic vaccines [35]. Although CpG ODN 1826 has been used as an adjuvant in animal studies of similar vaccines, we did not use it in the current study because it was not available to us for clinical use.

Our results, combined with existing evidence on the mechanism of CyaA-based vaccines, suggest that the CyaA portion of GTL001 binds to CD11b^+^ APC, delivering the partner HPV16 and HPV18 E7 antigens to the cytosol, where they are processed and then presented on MHC molecules. This results in an E7-specific CD8^+^ CTL response that can eradicate HPV E7-expressing tumors. The unconjugated vaccine probably failed to exhibit any anti-tumor activity because, due to the low amount of HPV E7 antigens, vectorization by CyaA and therefore dedicated delivery to APCs was needed to induce a sufficient immune response. These findings therefore highlight the potential of the CyaA platform for delivering antigens to APC and inducing antigen-specific CTL responses.

The goal of GTL001 is to treat HPV-infected patients before immunosuppression and viral escape mechanisms develop in the cervix and before high-grade lesions appear. In the current study, we confirmed that GTL001 adjuvanted with topical imiquimod cream induces T-cell-mediated immunity against HPV-infected cells, which is considered necessary for controlling existing infections and malignancies [5, 9]. GTL001 adjuvanted with 5% imiquimod cream has already been evaluated in a phase I clinical trial in women infected with HPV16 or HPV18 but with normal cervical cytology. The study showed that GTL001 + imiquimod was safe and well tolerated and that it induced a HPV16- and HPV18 E7-specific cellular immune response [20]. Because of the promising preclinical and phase I clinical results, the efficacy of GTL001 for preventing cervical cancer is currently being evaluated in a phase II multicenter trial in women infected with HPV16 or HPV18 who have normal cytology or mild abnormalities.

## References

[1] Bosch FX, Lorincz A, Munoz N, Meijer CJ, Shah KV. The causal relation between human papillomavirus and cervical cancer. Journal of clinical pathology. 2002;55(4):244–65. Epub 2002/03/29. 11919208 10.1136/jcp.55.4.244 PMC1769629

[2] Stanley M. Potential mechanisms for HPV vaccine-induced long-term protection. Gynecologic oncology. 2010;118(1 Suppl):S2–7. Epub 2010/06/04. 10.1016/j.ygyno.2010.04.002 20494220

[3] Frazer IH. Interaction of human papillomaviruses with the host immune system: a well evolved relationship. Virology. 2009;384(2):410–4. Epub 2008/11/07. 10.1016/j.virol.2008.10.004 18986661

[4] Hildesheim A, Herrero R, Wacholder S, Rodriguez AC, Solomon D, Bratti MC, et al Effect of human papillomavirus 16/18 L1 viruslike particle vaccine among young women with preexisting infection: a randomized trial. JAMA: the journal of the American Medical Association. 2007;298(7):743–53. Epub 2007/08/21. 10.1001/jama.298.7.743 17699008

[5] Stern PL, van der Burg SH, Hampson IN, Broker TR, Fiander A, Lacey CJ, et al Therapy of human papillomavirus-related disease. Vaccine. 2012;30 Suppl 5:F71–82. Epub 2012/12/05. 23199967 10.1016/j.vaccine.2012.05.091 PMC4155500

[6] Kyrgiou M, Koliopoulos G, Martin-Hirsch P, Arbyn M, Prendiville W, Paraskevaidis E. Obstetric outcomes after conservative treatment for intraepithelial or early invasive cervical lesions: systematic review and meta-analysis. Lancet. 2006;367(9509):489–98. Epub 2006/02/14. 10.1016/S0140-6736(06)68181-6 16473126

[7] Vrzackova P, Weiss P, Cibula D. Sexual morbidity following radical hysterectomy for cervical cancer. Expert review of anticancer therapy. 2010;10(7):1037–42. Epub 2010/07/22. 10.1586/era.10.89 20645693

[8] Wit EM, Horenblas S. Urological complications after treatment of cervical cancer. Nature reviews Urology. 2014;11(2):110–7. Epub 2014/01/30. 10.1038/nrurol.2013.323 24473416

[9] van der Burg SH, Melief CJ. Therapeutic vaccination against human papilloma virus induced malignancies. Current opinion in immunology. 2011;23(2):252–7. Epub 2011/01/18. 10.1016/j.coi.2010.12.010 21237632

[10] Vici P, Pizzuti L, Mariani L, Zampa G, Santini D, Di Lauro L, et al Targeting immune response with therapeutic vaccines in premalignant lesions and cervical cancer: hope or reality from clinical studies. Expert review of vaccines. 2016:1–10. Epub 2016/04/12. 10.1080/14760584.2016.1176533 PMC5152541 27063030

[11] Brun JL, Dalstein V, Leveque J, Mathevet P, Raulic P, Baldauf JJ, et al Regression of high-grade cervical intraepithelial neoplasia with TG4001 targeted immunotherapy. American journal of obstetrics and gynecology. 2011;204(2):169 e1–8. Epub 2011/02/03. 21284968 10.1016/j.ajog.2010.09.020

[12] Kawana K, Adachi K, Kojima S, Kozuma S, Fujii T. Therapeutic Human Papillomavirus (HPV) Vaccines: A Novel Approach. Open Virology Journal. 2012;6:264–9. Epub 2013/01/24. 10.2174/1874357901206010264 23341862 PMC3547358

[13] Stanley M, Pinto LA, Trimble C. Human papillomavirus vaccines—immune responses. Vaccine. 2012;30 Suppl 5:F83–7. Epub 2012/12/05. 23199968 10.1016/j.vaccine.2012.04.106

[14] Morrow MP, Yan J, Sardesai NY. Human papillomavirus therapeutic vaccines: targeting viral antigens as immunotherapy for precancerous disease and cancer. Expert review of vaccines. 2013;12(3):271–83. Epub 2013/03/19. 10.1586/erv.13.23 23496667

[15] Khallouf H, Grabowska AK, Riemer AB. Therapeutic Vaccine Strategies against Human Papillomavirus. Vaccines. 2014;2(2):422–62. Epub 2014/01/01. 10.3390/vaccines2020422 26344626 PMC4494257

[16] Tran NP, Hung CF, Roden R, Wu TC. Control of HPV infection and related cancer through vaccination. Recent results in cancer research Fortschritte der Krebsforschung Progres dans les recherches sur le cancer. 2014;193:149–71. Epub 2013/09/07. 10.1007/978-3-642-38965-8_9 24008298

[17] Simsova M, Sebo P, Leclerc C. The adenylate cyclase toxin from Bordetella pertussis—a novel promising vehicle for antigen delivery to dendritic cells. International Journal of Medical Microbiology. 2004;293(7–8):571–6. Epub 2004/05/20. 10.1078/1438-4221-00291 15149033

[18] Berraondo P, Nouze C, Preville X, Ladant D, Leclerc C. Eradication of large tumors in mice by a tritherapy targeting the innate, adaptive, and regulatory components of the immune system. Cancer research. 2007;67(18):8847–55. Epub 2007/09/19. 10.1158/0008-5472.CAN-07-0321 17875726

[19] Preville X, Ladant D, Timmerman B, Leclerc C. Eradication of established tumors by vaccination with recombinant Bordetella pertussis adenylate cyclase carrying the human papillomavirus 16 E7 oncoprotein. Cancer research. 2005;65(2):641–9. Epub 2005/02/08. 15695409

[20] Van Damme P, Bouillette-Marussig M, Hens A, De Coster I, Depuydt C, Goubier A, et al GTL001, a therapeutic vaccine for women infected with human papillomavirus 16 or 18 and normal cervical cytology: results of a phase 1 clinical trial. Clinical cancer research: an official journal of the American Association for Cancer Research. In press. 10.1158/1078-0432.CCR-16-0085 27252412

[21] Barber DL, Wherry EJ, Ahmed R. Cutting edge: rapid in vivo killing by memory CD8 T cells. J Immunol. 2003;171(1):27–31. Epub 2003/06/21. 12816979 10.4049/jimmunol.171.1.27

[22] Dadaglio G, Fayolle C, Zhang X, Ryffel B, Oberkampf M, Felix T, et al Antigen targeting to CD11b+ dendritic cells in association with TLR4/TRIF signaling promotes strong CD8+ T cell responses. J Immunol. 2014;193(4):1787–98. Epub 2014/07/16. 10.4049/jimmunol.1302974 25024388

[23] Schon MP, Schon M. Imiquimod: mode of action. The British journal of dermatology. 2007;157 Suppl 2:8–13. Epub 2007/12/11. 18067624 10.1111/j.1365-2133.2007.08265.x

[24] Feltkamp MC, Smits HL, Vierboom MP, Minnaar RP, de Jongh BM, Drijfhout JW, et al Vaccination with cytotoxic T lymphocyte epitope-containing peptide protects against a tumor induced by human papillomavirus type 16-transformed cells. European journal of immunology. 1993;23(9):2242–9. Epub 1993/09/01. 10.1002/eji.1830230929 7690326

[25] Preville X, Leclerc C, Ladant D, Timmerman B, inventorsRecombinant protein carrying human papillomavirus epitopes inserted in adenylate cyclase protein or fragment thereof. Therapeutic uses thereof2005.

[26] Lin KY, Guarnieri FG, Staveley-O'Carroll KF, Levitsky HI, August JT, Pardoll DM, et al Treatment of established tumors with a novel vaccine that enhances major histocompatibility class II presentation of tumor antigen. Cancer research. 1996;56(1):21–6. Epub 1996/01/01. 8548765

[27] Dadaglio G, Morel S, Bauche C, Moukrim Z, Lemonnier FA, Van Den Eynde BJ, et al Recombinant adenylate cyclase toxin of Bordetella pertussis induces cytotoxic T lymphocyte responses against HLA*0201-restricted melanoma epitopes. International immunology. 2003;15(12):1423–30. Epub 2003/12/03. 14645151 10.1093/intimm/dxg144

[28] Schlecht G, Loucka J, Najar H, Sebo P, Leclerc C. Antigen targeting to CD11b allows efficient presentation of CD4+ and CD8+ T cell epitopes and in vivo Th1-polarized T cell priming. J Immunol. 2004;173(10):6089–97. Epub 2004/11/06. 15528345 10.4049/jimmunol.173.10.6089

[29] Rechtsteiner G, Warger T, Osterloh P, Schild H, Radsak MP. Cutting edge: priming of CTL by transcutaneous peptide immunization with imiquimod. J Immunol. 2005;174(5):2476–80. Epub 2005/02/25. 15728450 10.4049/jimmunol.174.5.2476

[30] Adams S, O'Neill DW, Nonaka D, Hardin E, Chiriboga L, Siu K, et al Immunization of malignant melanoma patients with full-length NY-ESO-1 protein using TLR7 agonist imiquimod as vaccine adjuvant. J Immunol. 2008;181(1):776–84. Epub 2008/06/21. 18566444 10.4049/jimmunol.181.1.776 PMC2583094

[31] Hemmi H, Takeuchi O, Kawai T, Kaisho T, Sato S, Sanjo H, et al A Toll-like receptor recognizes bacterial DNA. Nature. 2000;408(6813):740–5. Epub 2000/12/29. 10.1038/35047123 11130078

[32] Kapsenberg ML. Dendritic-cell control of pathogen-driven T-cell polarization. Nature reviews Immunology. 2003;3(12):984–93. Epub 2003/12/04. 10.1038/nri1246 14647480

[33] Kawai T, Akira S. The role of pattern-recognition receptors in innate immunity: update on Toll-like receptors. Nature immunology. 2010;11(5):373–84. Epub 2010/04/21. 10.1038/ni.1863 20404851

[34] Fehres CM, Bruijns SC, van Beelen AJ, Kalay H, Ambrosini M, Hooijberg E, et al Topical rather than intradermal application of the TLR7 ligand imiquimod leads to human dermal dendritic cell maturation and CD8+ T-cell cross-priming. European journal of immunology. 2014;44(8):2415–24. Epub 2014/05/16. 10.1002/eji.201344094 24825342

[35] Gotovtseva EP, Kapadia AS, Smolensky MH, Lairson DR. Optimal frequency of imiquimod (aldara) 5% cream for the treatment of external genital warts in immunocompetent adults: a meta-analysis. Sexually transmitted diseases. 2008;35(4):346–51. Epub 2008/03/25. 18360317 10.1097/OLQ.0b013e31815ea8d1

